# Chronic multichannel neural recordings from soft regenerative microchannel electrodes during gait

**DOI:** 10.1038/srep14363

**Published:** 2015-09-24

**Authors:** Katherine M. Musick, Jacopo Rigosa, Shreya Narasimhan, Sophie Wurth, Marco Capogrosso, Daniel J. Chew, James W. Fawcett, Silvestro Micera, Stéphanie P. Lacour

**Affiliations:** 1 Bertarelli Foundation Chair in Neuroprosthetic Technology, Laboratory for Soft Bioelectronic Interfaces, Centre for Neuroprosthetics, Ecole Polytechnique Fédérale de Lausanne (EPFL), Lausanne, Switzerland; 2 Bertarelli Foundation Chair in Translational NeuroEngineering, Center for Neuroprosthetics and Institute of Bioengineering, Ecole Polytechnique Fédérale de Lausanne (EPFL), Lausanne, Switzerland; 3 The BioRobotics Institute, Scuola Superiore Sant’Anna, Pisa, Italy; 4 Centre for Brain Repair, University of Cambridge, UK

## Abstract

Reliably interfacing a nerve with an electrode array is one of the approaches to restore motor and sensory functions after an injury to the peripheral nerve. Accomplishing this with current technologies is challenging as the electrode-neuron interface often degrades over time, and surrounding myoelectric signals contaminate the neuro-signals in awake, moving animals. The purpose of this study was to evaluate the potential of microchannel electrode implants to monitor over time and in freely moving animals, neural activity from regenerating nerves. We designed and fabricated implants with silicone rubber and elastic thin-film metallization. Each implant carries an eight-by-twelve matrix of parallel microchannels (of 120 × 110 μm^2^ cross-section and 4 mm length) and gold thin-film electrodes embedded in the floor of ten of the microchannels. After sterilization, the soft, multi-lumen electrode implant is sutured between the stumps of the sciatic nerve. Over a period of three months and in four rats, the microchannel electrodes recorded spike activity from the regenerating sciatic nerve. Histology indicates mini-nerves formed of axons and supporting cells regenerate robustly in the implants. Analysis of the recorded spikes and gait kinematics over the ten-week period suggests firing patterns collected with the microchannel electrode implant can be associated with different phases of gait.

Peripheral nerve neuroprostheses aim at establishing a reliable communication link between axons and electrodes to repair, restore or circumvent neural functions after peripheral nerve disorders. Nerves host up to thousands of afferent and efferent fibers. Interfacing each of them with a corresponding electrode would achieve high selectivity, but this task is most likely unrealistic. To date, several implantable electrode designs are being pursued to reliably and selectively communicate with peripheral nerves^1^.

Devices in the form of a wire or a cuff can efficiently deliver electrical neuromodulation to the peripheral nerves. Such neuroprostheses are routinely used to treat chronic pain, sensory deficits and epilepsy^2^. Several research electrode designs are being explored to stimulate efficiently small nerves and nerve fibers, and restore motor and sensory functions^1^,^3^,^4^,^5^,^6^,^7^. Challenges lie in the selective stimulation of nerve fibers but also in the access and surgical placement of the electrode next to the nerve, especially small visceral nerves^8^.

Recording nerve activity is significantly more challenging given the extracellular neural signals are of a few microvolts and are monitored in the noisy physiological environment. The critical tradeoff lies between invasiveness and selectivity of the electrode interface^1^. Extraneural cuff electrodes completely encircle the nerve and contain a number of electrode sites on their inner surface, facing the nerve^9^. They are minimally invasive, but their recording selectivity is limited to large subgroups of axons that sit close to the nerve perimeter^10^,^11^. A flat-interface nerve electrode (FINE) is a cuff interface with higher selectivity^12^. After surgical positioning, the FINE device slowly reshapes the nerve from its natural elliptical cross-section to a flat, ribbon-like shape thereby giving access to those fascicles naturally lying more centrally in the nerve^13^. Penetrating electrodes embody the next level of invasiveness. Longitudinal intrafascicular electrodes (LIFE) are ribbon-like interfaces cross the nerve epineurium and are weaved parallel to the nerve fascicules^14^. Microwire arrays, slanted Utah arrays and transverse intrafascicular multichannel electrodes (TIME) penetrate the intrafascicular space to interface axons within the fascicules^15^, and offer high signal-to-noise ratio recordings. However, the quality of the recordings often degrades over time as foreign body reaction may trigger electrode encapsulation, and the nerve moves with respect to the electrode sites in chronic settings.

With these observations in mind, we have designed and manufactured a nerve implant, which exploits the natural properties of the peripheral nerve to regenerate and offers spatially distributed electrodes for efficient axon-electrode coupling.

When the nerve is sectioned, the nerve stumps may be bridged with a single or multilumen conduit to support axon regeneration. Cuff electrodes may be used as regenerative implants. Sieve electrodes, prepared with polymers or silicon^16^,^17^ offer greater electrode-axon proximity and enable efficient recording of the small neural signals. However the stability of their recordings depends on the distance between the electrode and the nearest axon’s node of Ranvier^18^.

Regenerative microchannel electrodes are essentially “long” sieve implants that host millimeter length of the nerve^19^,^20^,^21^. In this design, the nerve regenerates in parallel “mini-nerves” - bundles of axons accompanied with blood vessels in each microchannel. An electrode exposed within the microchannel can monitor the neural activity from the corresponding mini-nerve. Enclosing the axons in a microchannel made of an electrically insulating material artificially increases the resistance R_ecf_ of the restricted extracellular medium^22^. This results in a significant rise of the recordable extracellular amplitude (V_out_) of the action potential as


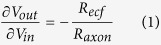


where V_out_, V_in_, R_ecf_, and R_axon_ are the extracellular potential, intracellular potential, extracellular resistance and axon resistance, respectively.

Microchannel electrodes have been previously tested^19^,^20^,^21^,^23^. The present study establishes a robust microfabrication method to produce the regenerative electrode implants using elastic materials and standard processing^24^ and provides more evidence about the possibility to record peripheral neural signals in freely moving animals and over time. We used the rat’s sciatic nerve model to evaluate the soft, regenerative microchannel electrode implant, and concurrently monitor over a period of 10 weeks neural activity and hindlimb kinematics of rats walking on a straight runway.

## Results

### An elastomeric regenerative electrode implant

**Figure 1:**
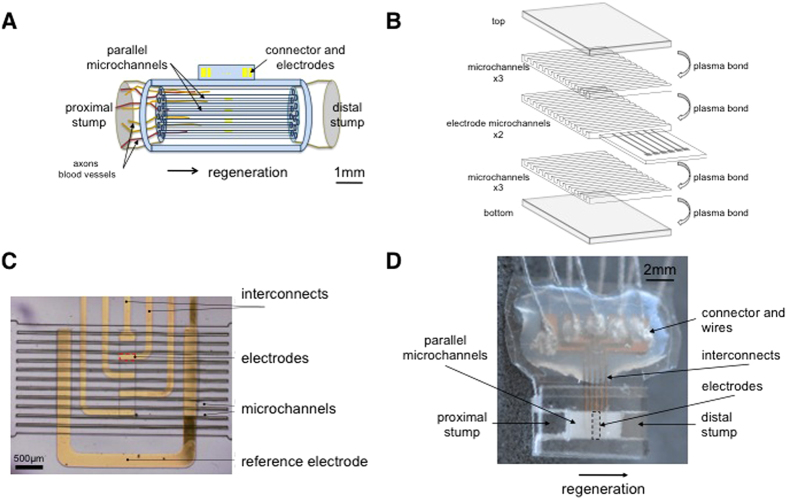
Regenerative microchannel electrode implant. (**A**) Concept drawing of the microchannel implant. The sciatic nerve is sectioned with each stump sutured into the implant, separated by an array of parallel microchannels. (**B**) Exploded, schematic view of the implant 14 elastomeric layers. The implant consists of 2 × 4 bonded rows of PDMS, each containing 10 microchannels. The microchannels are 110 μm × 120 μm, with a wall thickness of 50 μm. (**C**) Picture of the middle row of microchannels with integrated electrodes. (**D**) Picture of the complete elastomer-based microchannel electrode implant. The gold traces connecting to the electrodes extend out of the device and are interfaced to highly flexible wires with conductive paste.

Microchannel electrode implants were prepared with soft MEMS microfabrication techniques. A schematic of the implant is presented Fig. 1A. A detailed description of the materials and process flow is presented in the Methods section. Briefly, the implant consists of a block of parallel microchannels with a square (1.5 × 1.5 mm^2^) cross-section. The implant fabrication process includes three main steps (Fig. 1B): (1) molding of 8 PDMS microchannel layers against PDMS negative molds (each microchannel has a 120 × 110 μm^2^ cross-section and 4 mm length; there are 12 parallel microchannels per layer), (2) patterning of two thin-metal film microelectrode arrays embedded in PDMS membrane (Fig. 1C), and (3) alignment and plasma-bonding of the 12 elastomeric layers. The middle two layers each contain five thin-film electrodes embedded in and positioned at the mid-point of the corresponding five microchannels. Each end of the implant is formed of a hollow macroscopic tube, large enough to host the nerve stump (Fig. 1A). Thin gold film interconnects extend out of the implant to contact pads, that are connected with a conductive paste to wires leading to a headstage connector. These connections are further insulated and secured with a layer of silicone (Fig. 1D).

### Regeneration through the michrochannel electrode implant

**Figure 2:**
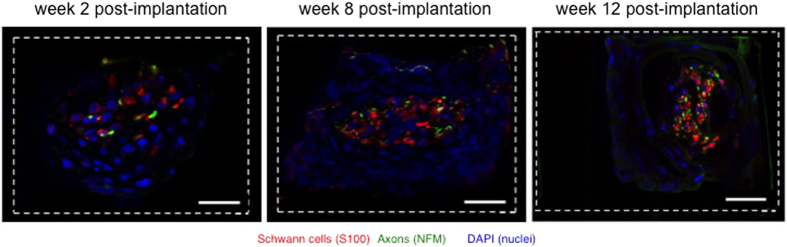
Representative images of the axonal regrowth through the implant in three distinct microchannels and at three time points, showing axons (green), Schwann cells (red) and cell nuclei (blue). Regeneration of myelinated axons in the distal portion of the implant can be seen as soon as two weeks post-implantation with further qualitative increase over the next two months. (d) The same as in (b) with DAPI shows that channels were filled with cells. (**A**) 2 weeks, (**B**) 8 weeks, and (**C**) 12 weeks post-implantation. Scale bar of 20 μm; dotted line overlays the microchannel geometrical contour.

Morphological evaluation was performed on the tissue-filled devices explanted after the neural recording sessions at three distinct timepoints, i.e. at 2, 8 and 12-week post-implantation. Semi-thin sections were cut to examine the tissue removed from the center (mid-length) of the device. Histology images confirmed the sciatic nerve regenerates through the microchannel implants, forming an artificial fascicular structure (Fig. 2), and axons have reached the mid-length of the mutilumen conduits at week 2 post-implantation. Samples preparation for immunohistochemistry proved particularly challenging as tissue fixation and subsequent sectioning within the elastomeric scaffold was difficult. This prevented us from conducting quantitative evaluation of cellular regeneration through the microchannels. Nonetheless, across all 12 animals (n = 4 at each time point), we observed that most microchannels were filled with a miniature nerve fascicle including myelinated and unmyelinated axons, Schwann cells, and blood vessels surrounded by connective tissue (Fig. 2). In each microchannel, a sheath of collagen formed along the walls of the channel. This ring is thin during the early period of regeneration, surrounding a dense collection of up to >100 myelinated axons (Fig. 2C, 3-month post-implantation).

### Microelectrode impedance

On a selected regenerative implant, the impedance of seven electrodes was measured at 1 kHz in phosphate buffer solution against an Ag/AgCl wire at room temperature, before implantation and after explantation. The average impedance increased from 300 ± 171 kΩ at an angle of −24 ± 4° to 395 ± 61 kΩ at an angle of −42 ± 12° (mean ± std dev.) after 7-week implantation. This impedance increase over weeks of implantation is not unusual in microfabricated implants^25^,^26^,^27^.

### Chronic recordings of neural activity in walking animals

**Figure 3:**
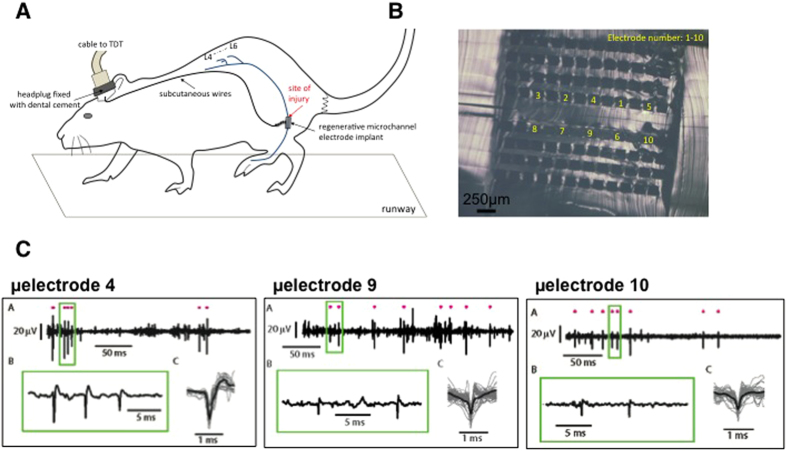
Sciatic nerve activity during walking on the runway. (**A**) Schematic of the experimental set-up with the recording neuroprosthesis. (**B**) Cross-sectional photograph of the regenerative PDMS microchannel array highlighting the ten “electrode” channels, labeled 1 to 10 in the two middle rows of the implant. (**C**) Representative recordings collected from three microelectrodes (4, 9 and 10). For each electrode, raw data are presented over 250 ms (top) and 20 ms (bottom left) periods. Single spike activity with overlaid average waveform is shown on the right. All recordings were performed at 6 weeks post-implantation.

Four animals were trained to walk on a straight runway before being implanted with the regenerative neuroprosthesis. After implantation, the animals were given three weeks rest to allow full recovery. From week 3 to week 10, we performed weekly recordings of neural data and kinematic data while the rats were performing the runway task. The recording setup is shown in Fig. 3A. The implant resides on the sciatic nerve and the wiring runs subcutaneously up to the headplug. The latter is then connected to the recording setup via a cable, which is easily detachable and does not interfere with the rat’s ability to walk down the runway. The kinematic behavior was filmed with 100-Hz cameras, and the video files and neural data streams were synchronized with a trigger to correlate step timing and spike events recorded through the ten microelectrodes (Fig. 3B).

Three representative data streams generated during the runway trials, and obtained from a rat at 6 weeks post-implantation, are shown Fig. 3C. All traces include for each electrode the raw data, a spike overlay and an spike average. The microelectrodes allow for the detection of action potentials that clearly lie beyond the noise level, and demonstrate a characteristic spike shape.

**Figure 4:**
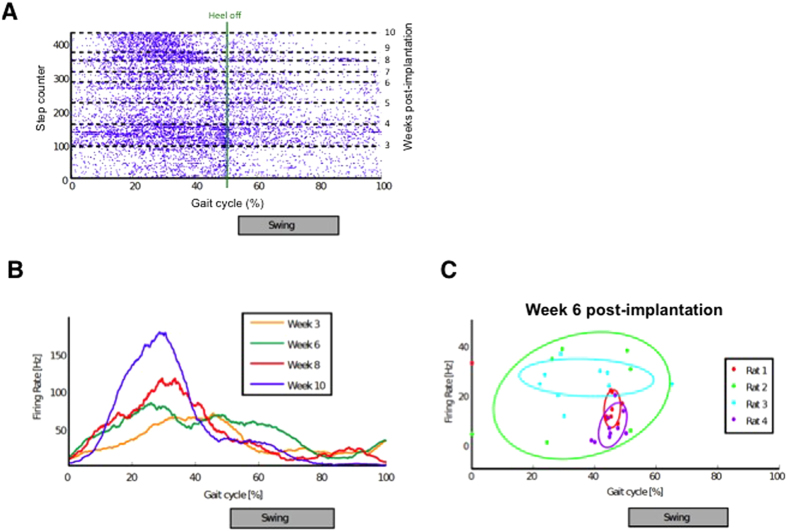
Neuronal activity from freely moving rats. (**A**) Raster plots of single-unit activity over the gait cycle obtained from weekly recordings in one rat. Raw data is collected from all ten electrodes in the implant. (**B**) Firing rate as a function of gait cycle computed from recordings from all ten electrodes at weeks 3, 6, 8, and 10 in one rat. (**C**) Firing rate from fibers regenerating within each electrode microchannel (n = 10) in each of the four rats and collected at week 6 post-implantation.

Heel-off events were retrieved from the videos of each individual trial, so that it was possible to correlate them with the neural firing activity. The multiunit activity recorded across all ten-electrode channels in a rat and from week 3 to week 10 post-implantation is shown via a raster plot (Fig. 4A). Each line on the raster plot corresponds to a single step, with the heel-off event centered at 50% of the gait cycle. Representation in gait percentage (as opposed to in seconds) allows for a comparison between steps despite variations in stepping speed. The average firing rate over the gait cycle was calculated and is displayed for four representative weeks for one rat (Fig. 4B). A general increase in firing rates and narrowing of the peak each week is shown, suggesting an improvement in regeneration and restored neural function over time.

In a healthy rat, extension muscles e.g. the tibialis anterior (TA), are activated when the foot leaves the ground, while flexion muscles e.g. the gastrocnemius (GA) are activated during the stance phase, directly before the heel-off event. Analysis of the firing patterns recorded with all 10 × 4 electrodes revealed the firing rate peak may shift relative to the heel-off event. Overall, the firing activity was mostly observed between foot-strike and heel-off (stance phase of the gait). However in some rats, we also detected two different categories of firing patterns. Firing peaks were seen either before heel-off events, suggesting motor fibers activation produced leg flexion, or after foot-strike events, when motor fibers triggered leg extension.

Figure 4C summarizes the position of the firing rate peaks during the gait cycle in all four evaluated animals (n = 4 × 10 electrodes total). Data are shown at week 6 post-implantation. In rats 1 and 4, regenerated neurons predominately fired during leg extension. A wider variety of activities was captured with Rats 2 and 3, suggesting that fascicles destined to a wider range of muscles regenerated into the electrode-containing channels of these devices.

## Discussion

### The soft microfabricated implant for regenerative nerve is a stable neural interface *in vivo*

We successfully implanted regenerative microchannel electrode implants made of silicone rubber and elastic thin-metal film electrodes chronically and collected electrophysiological data from the regenerating nerves *in vivo*. Neural activity was recorded from microelectrodes embedded in the silicone microchannels within the noisy environment of a freely walking animal.

The implant is entirely prepared with soft and elastic materials and integrated using standard microfabrication process. The mechanical properties of these elastomeric implants are close to those of the sciatic nerve, with an elastic modulus of 1.2 MPa for the supporting PDMS compared to a few MPa for the repaired peripheral nerve^28^,^29^. Upon surgical implantation, the soft implant is easily manipulated and sutured in-between the nerve stumps (Fig. 1A). Over the course of the study and after optimization of the animal headplug and length of the subcutaneous wires connecting the plug to the regenerative implant, we did not observe electrode failure in any of the implanted devices.

The microchannel electrode design offers stable access to regenerated nerve fibers across the whole nerve section. The three-dimensional multilumen construct guides regenerating axons and Schwann cells along the longitudinal channels and towards the embedded microelectrodes thereby facilitating a stable electrical neuron-electrode interface. The narrow and long channels avoid the need for electrode-node of Ranvier proximity^22^. The high quality, multichannel recordings obtained during our three-month feasibility study are encouraging results to pursue the development and implementation of the regenerative microchannel implant.

### The microchannel electrodes enable high quality recordings leading to useful electrophysiological data

Neural activity was successfully recorded from four animals walking along the runway from week 3 post-implantation then over several weeks. The firing of action potentials was observed to be synchronous with the gait cycle of the animal, with an increase of the spike rate over time, probably correlated to the maturation of the regenerating nerve and axon myelination. These findings also indicate that axons regenerated through the 4 mm long microchannels.

Distinct firing patterns with the gait cycle were observed across the four animals. In the case of rats 2 and 3, the firing activity from all channels is spread across the gait cycle indicating a mixed distribution of the axons in the multilumen implant. In contrast, in rats 1 and 4, the firing predominantly occurs during leg extension, which suggests most electrode microchannels carry axons reinnervating the TA. In our current surgical procedure, only two sutures are used to secure the implant at each nerve stump. It is therefore possible that predominately one fascicle has regenerated through the electrode-containing channels. Future work on this class of devices should therefore aim at (1) optimizing the surgical techniques to better control, at the time of implantation, the location of the fascicles relative to the electrode layers and (2) placing the electrodes on two non-adjacent layers of the device to increase the likelihood of a wider variety of signals obtained from a single rat.

### Potential use of soft regenerative electrodes for neuroprosthesis control

During our experiments, the high selectivity of the soft regenerative electrodes allowed the extraction of multi-unit signals recorded with freely moving animals performing gait tasks. These results open up very nice opportunities to develop neuroprosthesis control algorithms based on the processing of these signals. The microchannel interface opens up the possibility to identify individual axons or small groups of axons within the channels. Spike-based analysis can provide richer and more usable information than “cumulative” (global) signals as shown in^30^ for peripheral nerves. Therefore, these signals could be used to control artificial limbs improving and extending what was already shown in amputees by off-line processing peripheral neural signals^31^. It could be also possible to gather information about the limb (e.g., gait phases) during specific tasks (in particular locomotion) to develop state-based control algorithms.

Finally, the long-term reliability of the neural recordings shown in our study clearly illustrates the potential of microchannel electrode-based implants and indicates the possibility to perform closed-loop control of organ functions, e.g. bladder control^20^.

## Conclusion

In summary, regenerative microchannel electrode implants offer unique, selective recording capabilities, unmet today with alternative designs. Their MEMS-based microfabrication offers an opportunity for implant batch-fabrication with high reproducibility, a mandatory step for *in vivo* experimentation in animal-models and potential clinical translation.

We conducted a trial run on in-channel stimulation of the regenerated fibers at 8 weeks post-implantation and observed activation of both TA and GA muscles using one or simultaneously two microchannel electrodes. Local stimulation (in the microchannels) of the nerve fibers will next be evaluated aiming at optimizing electrode distribution in the implant and stimulation parameters for selective activation of the fibers and recruitment of the muscles.

In a previous study, we showed the microchannel interface supports functional axon regeneration over an extended period of nine months in rats^32^. Despite a reduction of axon numbers per microchannel with time, this suggests a long time window for repeated recording of the sciatic nerve activity in order to assess the sensory-motor activity of the regenerating nerve.

With these unique properties, the regenerative microchannel electrodes offer an exciting avenue for motor control and sensory feedback in closed-loop neuroprosthetic systems.

## Methods

### Implant microfabrication

Microchannel electrode implants were prepared with soft MEMS microfabrication techniques. The process began with the patterning of a linear array of microchannels in silicon using deep reactive ion etching. The array consists of 12 parallel channels of 110 μm height, 120 μm width, and 4 mm length. After silanization of the Si wafer, (trichloro(1H,1H,2H,2H-perfluorooctyl)silane (Sigma Aldrich) deposited in vapor phase), a ~1 cm thick layer of PDMS (Sylgard 184, Dow Corning, 10:1 prepolymer:curing agent) is casted on the Si patterns and peeled-off to form a negative replica. Next and after another silanization step, the microchannel layers are prepared by spin-coating PDMS (Sylgard 184, Dow Corning, 10:1 prepolymer:curing agent; 575 rpm for 30 s) on the PDMS mold followed by curing in an oven at 80 °C for 2 h.

Microelectrodes and interconnects were prepared on a flat, 150 μm thick PDMS membrane spun on a water-soluble poly(4-styrenesulfonic acid)-coated silicon wafer. A bi-layer of Cr/Au (5/30-nm thick) was thermally evaporated onto the PDMS through a polyimide shadow mask. The evaporated metal spontaneously forms a micro-textured structure that yields a highly flexible and stretchable conductive film^24^,^33^. The thin-film interconnects were encapsulated with a plasma-bonded 30 μm-thick membrane of PDMS leaving 100 μm × 200 μm rectangular electrode sites open (Fig. 1C).

Next, the implant was assembled by stacking the various layers (Fig. 3B). A microchannel layer was peeled off of the mold. Both this layer and the electrode layer (still on the wafer) were exposed to air plasma (25 s, 29 W), and aligned with a custom-built apparatus. This process was repeated to achieve four microchannel layers on top of the electrodes. Then the entire stack was lifted-off of the wafer by dissolving the PSS in deionized water, rinsing with IPA, and drying. Two copies of this electrode-microchannel stack were made then plasma-bonded backside-to-backside to form the device (Fig. 1D).

A U-shaped section (for the nerve stumps) was then cut out of each end of the stack of channels, leaving a wall 0.5-mm wide on each side. On the top and bottom of the stack, a sheet of PDMS (0.5-mm thick) was bonded similarly and completed the implant (Fig. 1D).

Next, the electrodes were interfaced to a headstage connector (A19923-001, Omnetics) with insulated wires. Electrical contact between the contact pads on the PDMS and the wires with silver paste. These connections were encapsulated with silicone glue (Fig. 1D). Twelve implants were prepared and wired.

### *In vivo* implantation

All surgical procedures were performed in accordance with Swiss federal legislation and under the guidelines established at EPFL. Local Swiss Veterinary Offices i.e. the Swiss Service de la consummation et des affaires vétérinaires du Canton de Vaud, Switzerland, approved all the procedures.

Adult male Lewis rats (250 grams, N.23, Charles River, UK) were used throughout this study. Prior to surgery, all implants were sterilized in 70% ethanol for 30 min. Surgeries were performed under inhalant anesthesia of 2% isofluorane in 2 L/min oxygen. The left sciatic nerve was exposed at the thigh and freed from surrounding tissues, from the sciatic notch to the knee. The sciatic nerve trunk was transected, and each nerve stump was sutured through the epineurium into the proximal and distal ends of the PDMS device with three 10-0 ethicon sutures. The stumps are separated by 4 mm, with the microchannel array positioned in this gap. Prior to implantation, implants were filled with saline solution. After implanting the device, the muscle and skin were re-approximated and sutured.

An ultra-flexible wire bundle connecting the contact pads on the implant to the Omnetics headstage was threaded subcutaneously along the back of the animal and exteriorized behind the head. Three small screws were attached to the skull in order to anchor the headstage, which was subsequently fixed to these screws with dental cement.

### Histology

For histological evaluation, animals under terminal pentobarbital anesthesia were transcardially perfused with 0.9% saline solution followed by 4% paraformaldehyde in 0.01 M phosphate buffer solution (PBS), and the implant together with a section of sciatic nerve attached to each end were harvested along with the contralateral sciatic nerve. The nerves were submerged in the paraformaldehyde solution for 24 h for further fixation.

For immunohistochemistry, the tissue was then transferred to a 30% sucrose solution for 3 days. The nerve-filled PDMS scaffold with proximal and distal stumps attached were embedded in OCT before being cryosectioned. The block was sectioned transversally at 100 μm thickness. The sections were stained against axons (NFM, millipore, MAB 5262), Schwann cells (S-100, Sigma-Aldrich, S2644) and DAPI. The sections were thawed and washed with 0.1 M PBS, followed by 90 min of blocking at room temperature with goat serum (1:25 dilution) and Triton-X (0.5%) in 0.1 M PBS. Wash buffer consisted in a solution of 5% Triton-X in 0.1 M PBS.

The primary incubation was done overnight at 4°C NFM and S100 were used with dilution of 1:400 and 1:250, respectively, in blocking solution. Secondary antibodies used were Alexa 488 (1:400) and Alexa 647 (1:250) in wash buffer for 90 min at room temperature. The sections were sealed with fluoromount and cover-slipped while still hydrated in order to avoid bubble formation. Images and Stacks of up to 40 μm were were collected with a confocal scanning microscope (Zeiss, LSM 700) under 20× magnification.

### Neural recordings in freely moving animals

For 3 weeks prior to the implant surgery, the rats were trained to walk down a 1 m long runway without stopping between steps. Starting at day 8 post-surgery, the rats performed this runway task on a weekly basis. The neural activity was amplified and recorded using a Tucker Davis Technologies system (TDT PZ2) at the sampling frequency of 25 kHz. Two high-definition cameras (Vicon, Oxford, UK) were used to record at 100 Hz the video of the task from both sides of the runway in order to extract information about the rat hind limb joint kinematics and the timing of the gait cycle for every step during each trial. The synchronization of TDT and Vicon data streams was possible offline by means of a trigger signal.

### Signal processing of the recorded data

Peripheral extracellular recordings have first been filtered in a wavelet domain^30^ in order to enhance the spiking activity with respect to the background noise. Wavelet denoising relies on the idea that a noisy signal can be better represented in an orthogonal time-frequency domain where a potential separation between noise and signal is possible. The noise is then reduced by coefficient thresholding in that domain, and the denoised signal is then reconstructed.

Spike events have been identified from the filtered signals by means of thresholding: if a sample of the denoised signal exceeds a voltage threshold identified as four times its standard deviation, then a spike occurrence is considered. The multiunit sequence of spikes was then aligned to the time of the heel-off event, readily available from the video recordings.

In order to compare steps of different duration, the time between two heel-off occurrences has been normalized and renamed gait cycle percentiles (Fig. 4A). The average number of spikes across all channels that occurs in a specific percentile of the gait cycle has been estimated by means of a smoothing window of width 5 percentiles (Fig. 4B). In addition to this, in order to distinguish the overall activity of each channel for the whole cohort as a function of the gait cycle percentiles in a glance, the firing rate peak of multiunit activity separated channel by channel has been scattered with respect to the relative percentile of the gait cycle (Fig. 4C).

Furthermore, we defined a signal-to-noise ratio (SNR) parameter to measure the quality of recorded signal as the ratio of the standard deviation of action potentials over the standard deviation of the overall signal: the higher this value, the easier the spike detection.

## Additional Information

**How to cite this article**: Musick, K. M. *et al.* Chronic multichannel neural recordings from soft regenerative microchannel electrodes during gait. *Sci. Rep.* **5**, 14363; doi: 10.1038/srep14363 (2015).
